# Teaching Seasoned Doctors New Technology: An Intervention to Reduce Barriers and Improve Comfort With Clinical Ultrasound

**DOI:** 10.7759/cureus.17248

**Published:** 2021-08-17

**Authors:** Sarah K Kennedy, Taylor Duncan, Audrey G Herbert, Loren K Rood, Matt A Rutz, Gregory S Zahn, Julie L Welch, Frances M Russell

**Affiliations:** 1 Emergency Medicine, Indiana University School of Medicine, Indianapolis, USA; 2 Emergency Department, St. Elizabeth Hospital, Edgewood, USA

**Keywords:** faculty development, ultrasound, clinical ultrasound, ultrasound curriculum, point-of-care ultrasound, ultrasound workshop

## Abstract

Introduction

Although clinical ultrasound (CUS) is a core skill that is a requirement for emergency medicine (EM) residency graduation, only a fraction of EM practitioners who trained prior to this requirement are certified in CUS. The objective of the study was to implement a CUS workshop for practicing EM physicians, identify barriers to utilization, and assess comfort with the machine, obtaining and interpreting images, and incorporating CUS into clinical practice.

Methods

This was a prospective descriptive cohort study of EM physician faculty who participated in an interactive 5-hour CUS workshop intervention that introduced four core CUS modalities via didactics and hands-on scanning stations. Pre- and post-surveys were administered to identify barriers to utilization and assess perceived comfort with CUS using a 5-point Likert scale. Results were analyzed using Fisher’s exact and paired t-tests.

Results

Thirty-five EM physicians participated with a 100% survey response rate. Only five of the physicians were ultrasound certified at the time of the workshop. On average, physicians were 16 years post-residency. Prior to the workshop, 29% had minimal ultrasound experience and 43% had not performed more than 50 ultrasounds. In the pre-course survey, every physician expressed at least one barrier to CUS utilization. Post-workshop, physicians felt significantly more comfortable using the ultrasound machine (p=0.0008), obtaining and interpreting images (p=0.0009 and p=0.0004), and incorporating CUS into clinical practice (p=0.002).

Conclusion

This workshop is an effective tool to expose practicing physicians to core concepts of CUS, improve their comfort level, and reduce barriers to ultrasound utilization.

## Introduction

The incorporation of clinical ultrasound (CUS), or point-of-care ultrasound (POCUS), into patient care and academic settings is a steadily growing practice. The use of CUS in the emergency department (ED) is associated with decreased time to definitive diagnosis, decreased time to disposition, decreased radiation exposure, and increased patient satisfaction [1-7]. Additionally, ultrasound to facilitate procedural guidance has been found to increase first pass success and decrease complications [8]. In academic settings, CUS is being incorporated into every stage of medical training.

Ensuring that emergency medicine (EM) physicians are trained to utilize CUS has become a priority in the past decade. In 2012, the Accreditation Council for Graduate Medical Education (ACGME) deemed CUS a core skill and competency for EM residency graduates [9]. Therefore, all EM residents since that time have been required to be competent in the core modalities of CUS including cardiac, aorta, Extended Focused Assessment with Sonography in Trauma (EFAST), and obstetric (OB) [10]. In addition, over 100 emergency ultrasound fellowships are available in the U.S. for advanced training post-residency [11].

However, survey-based literature suggests that only a fraction of EM physicians who trained prior to 2008 are certified in CUS and incorporate it into daily practice [10, 12, 13]. In academic settings, only half of EM training programs have more than 50% of their faculty physicians certified to perform and teach CUS [14]. A needs assessment in our own academic department found that only 33% of our EM faculty physicians were ultrasound certified, primarily through residency training, an ultrasound fellowship, or a registered diagnostic medical sonographer program. In academic settings, the consequences include the supervision of learners whose comfort and skills with CUS may exceed that of the supervising physician. Therefore, additional training options are needed to reach all EM faculty physicians and practicing physicians, especially those who completed EM residency training prior to the ACGME requirements.

This study fills a gap in the ultrasound literature by offering an introductory CUS course to practicing EM physicians based on the American College of Emergency Physicians’ (ACEP) “practice-based pathway,” which is used worldwide as a standard for clinical ultrasound programs [7, 15, 16]. EM physicians have previously identified barriers to CUS utilization, including a lack of confidence and comfort in operating the machine, obtaining images, interpreting images within the core modalities, and documentation of examinations [17-20]. While there are examples of train-the-trainer, ultrasound workshops in critical care settings, targeted organ pathology, and simulation cases, there is a paucity of CUS educational approaches that specifically address these barriers and target the needs of non-certified practicing EM physicians [21-25].

The purpose of this study is to describe the implementation and evaluation of an educational intervention to introduce practicing EM physicians to the core modalities of CUS, identify barriers to ultrasound utilization, and assess physician comfort with the ultrasound machine, obtaining and interpreting images, and incorporating ultrasound into clinical practice.

## Materials and methods

Study design

This was a prospective descriptive cohort study of Emergency Medicine (EM) faculty in an academic EM department covering three urban teaching hospitals that serve a large medical school and EM residency program. This target audience included practicing EM physicians with varying CUS experience, therefore no prerequisite knowledge or skill were required to participate. The CUS workshop intervention was a voluntary professional development opportunity, offering continuing medical education (CME) through the institution. A recruitment email invitation was sent via a departmental listserv to all EM physicians describing the workshop and study. Two back-to-back workshop days were scheduled, with one session in the afternoon and the other in the morning. As the emergency department (ED) cannot go unstaffed, we accommodated flexible scheduling for faculty. We capped each workshop at 20 participants per day. This study was granted exempt status by the Institutional Review Board at Indiana University.

Clinical ultrasound workshop content

The intervention consisted of a 5-hour workshop that included didactic lectures and hands-on ultrasound scanning sessions. Materials for this workshop were developed and implemented following the workshop schedule, educational activities, and objectives found in Appendix A. The interactive content covered four core ultrasound modalities (i.e., EFAST, cardiac, aorta, and OB ultrasound) as specified by the ACEP “practice-based pathway” for currently practicing providers and served as an introduction or refresher course [7]. The practice-based pathway encourages formal lectures and hands-on proctored scanning, both of which are incorporated learning methods within the workshop. This educational approach incorporates elements from Kolb’s experiential learning theory, which accommodates different stages of learner knowledge with mixed learning styles and preferences as well as Gagne’s instructional design model, which serves as a template for procedural instruction [26, 27]. The workshop emphasized hands-on practice using an equal ratio of lecture to hands-on scanning time to offer real-time application of knowledge just learned and one-on-one feedback from instructors. The workshop content also provided information about basic ultrasound physics and how to use the ultrasound machines at our institution (i.e., knobology). In addition, we delivered a lecture titled SonoSaves to highlight interesting and time-critical cases (i.e., great saves) in which CUS was instrumental during the care of patients.

Instructors

Instructors for the workshop included five EM physician faculty from the ultrasound division with ultrasound training and certification. They prepared and delivered lectures in PowerPoint format, with an emphasis on dynamic clips and images rather than text. All ultrasound images and movie clips were original scans captured by the ultrasound division. To ensure high-yield content and keep the participants engaged, lectures were kept to 20 minutes at maximum length with no more than one hour of continuous lecturing. After each lecture topic, instructors moved to hands-on scanning stations where the participants practiced setting up the machine, selecting a transducer, acquiring images, and saving images.

Hands-on scanning stations 

We created four stations for hands-on scanning with one instructor and five learners per station. Each station consisted of a standardized patient model for live scanning, a bed, and a portable ultrasound machine with both curvilinear and phased-array probes. We used Zonare ZS3 machines (Mindray North America, Mahwah, NJ, USA), as those are the machines available in our emergency departments. Each station also had ultrasound gel, sheets, towels, and probe cleaning wipes. With an ultrasound-trained and certified faculty instructor at each station, this provided real-time, expert feedback with every scan. Standardized patient models were recruited through the institution simulation center and from residents and students on their ultrasound rotation.

Evaluation strategy

Pre- and post-workshop surveys were utilized to evaluate the intervention. A survey design was modeled after Kirkpatrick’s level one evaluation of the learner’s reaction or perception, including perceived comfort and barriers, as frequently used in ultrasound literature [17, 18, 20, 28]. The pre-course survey (Appendix B) was distributed to the participants and collected at the beginning of the CUS workshop. This survey was divided into three parts with seven total questions. The first part included demographics, a self-assessment for the level of CUS experience, and an estimated number of scans performed. The second part evaluated perceived barriers to ultrasound utilization in the ED. The third portion used a 5-point Likert scale to assess physician comfort with using the machines, obtaining images, interpreting images, and incorporating ultrasound into clinical practice. The post-course survey (Appendix C) was administered to the physicians at the end of the workshop. This mirrored the pre-course survey, which allowed for direct before and after comparison of perceived barriers and comfort with aspects of CUS. 

Data analysis

Data were managed using a secure web-based application. Participant characteristics and perceived barriers were expressed as frequency counts and percentages. Comfort levels were reported as the mean of Likert scale responses. Differences between pre- and post-survey responses were analyzed using Fisher’s exact and paired t-tests. Statistical significance was accepted for p<0.05.

## Results

A total of 35 EM physician faculty members attended the workshop intervention. Pre- and post-surveys were completed with a 100% response rate. On average, physicians were 16 years post-residency graduation, ranging from five to 34 years. Prior to the ultrasound workshop, 29% had minimal ultrasound experience, 43% had not performed more than 50 ultrasounds, and five were previously ultrasound certified (Table 1). Instructors included five ultrasound-trained and certified faculty members from the division of ultrasound.

**Table 1:** Emergency Medicine Physician Faculty Participant Characteristics, n = 35

Characteristics		n (%)
Male		23 (66%)
Number of Years since Graduation		
	<10 years	5 (14.3%)
	10-15 years	12 (34.3%)
	16-20 years	10 (28.6%)
	>20 years	8 (22.9%)
Number of Prior Ultrasounds		
	<25 ultrasounds	4 (11.4%)
	26-50 ultrasounds	11 (31.4%)
	51-100 ultrasounds	5 (14.3%)
	>100 ultrasounds	15 (42.9%)
Level of Ultrasound Experience		
	None	0 (0%)
	Little to Some	10 (28.6%)
	Moderate	20 (57.1%)
	Large	5 (14.3%)

The pre-course survey identified multiple barriers to ultrasound utilization, with every physician participant expressing at least one barrier. The majority of EM physicians perceived that ultrasound was too time-intensive to perform clinically (60%), were not confident in their ultrasound skills (60%), and did not know how to submit images for quality assurance (80%). Fifteen of 35 (43%) did not know how to use the ultrasound machine, while 34% of participants did not know how to interpret CUS images. Four of 35 (11%) did not see the utility in performing CUS (Table 2).

**Table 2:** Emergency Medicine Physician Faculty Perceived Barriers to Utilizing Clinical Ultrasound, n = 35 ^a^Significance p < 0.05 ^b^QA = quality assurance

Barrier to Utilizing Clinical Ultrasound	Pre-Workshop, n (%)	Post-Workshop, n (%)	p-values^a^
Takes too much time	21 (60%)	20 (57%)	p = 0.811
Don’t know how to use the machine	15 (43%)	3 (8.6%)	p = 0.0008
Don't know how to interpret images	12 (34%)	6 (17%)	p = 0.103
Comfort with personal skills	21 (60%)	18 (51%)	p = 0.480
Don't see the utility	4 (11%)	1 (2.8%)	p = 0.169
Lack of machine availability	4 (11%)	0 (0%)	p = 0.040
Don't know how to submit images for QA^b^	28 (80%)	4 (11%)	p < 0.0001
None	0 (0%)	6 (17%)	p = 0.010

When comparing perceived barriers pre- and post-workshop, there was a significant decrease in physicians who reported the barriers of not knowing how to use the ultrasound machine or submit images for quality assurance (p=0.0008 and p<0.0001, respectively). In addition, significantly more physicians identified no barriers to ultrasound utilization after the workshop (p=0.01). Of the remaining barriers, there was a trend towards fewer perceived obstacles to using ultrasound post-workshop (Table 2).

Physician comfort in using CUS was reported on a 5-point Likert scale. Pre-course, physicians were least comfortable interpreting images (2.83/5.0), followed by using the ultrasound machine (2.89/5.0), obtaining images (2.94/5.0), and incorporating ultrasound into clinical practice (3.14/5.0). Post-workshop, physicians felt significantly more comfortable using the ultrasound machine (p=0.0008) and six participants identified no barriers to ultrasound utilization (p=0.01) (Table 2). Participants also reported improved comfort with obtaining and interpreting images (p=0.0009 and p=0.0004, respectively), and incorporating CUS into clinical practice (p=0.002) (Table 3).

**Table 3:** Emergency Medicine Faculty Reported Comfort in Utilizing Clinical Ultrasound, n = 35 ^a^Scoring was based on a 5-point Likert scale (1 = strongly disagree to 5 = strongly agree) ^b^Significance p < 0.05

Comfort with	Pre-Workshop Mean^a^	Post-Workshop Mean^a^	p-values^b^
Ultrasound Machine	2.89	4.76	p = 0.0001
Obtaining images	2.94	3.74	p = 0.0009
Interpreting images	2.83	3.68	p = 0.0004
Incorporation into daily practice	3.14	3.86	p = 0.0023

## Discussion

The aims of this study were to identify perceived barriers to ultrasound utilization and assess comfort with ultrasound among EM physicians before and after a focused ultrasound workshop. We found that after a dedicated 5-hour intervention, these aims were effectively accomplished through lectures and hands-on scanning. The hands-on component was critical for physicians to practice using the machine, acquire and interpret images, as well as submit CUS scans for quality assurance. This intervention was found to significantly improve EM physician comfort in each of these categories, as well as incorporating ultrasound into clinical practice. After the workshop, EM physicians also identified significantly fewer barriers to using CUS.

Clinical ultrasound (CUS) is an actively growing field in EM and other specialties [17, 18, 29], and utilization of CUS is associated with improved outcomes and increased patient satisfaction [1-6]. For these reasons, increasing EM physician faculty comfort and confidence with CUS is critical for managing patients and supervising learners. While a minority of EM physicians who trained prior to 2008 are certified in CUS, there is a need for studies on how to effectively train these physicians in CUS, whether as an educational program or refresher course [10, 30]. Graglia et al. provide evidence that physician comfort with using CUS is an important outcome measure to address in training physicians [17]. Our workshop provides additional evidence as an effective educational tool to expose seasoned EM physicians to core concepts of CUS and increase their comfort with this technology.

We were able to identify several perceived barriers that physicians face in utilizing bedside ultrasound in the clinical environment and saw a reduction after the workshop in these barriers including knowing how to use the machine and submitting images for quality assurance. Prior literature has identified barriers to ultrasound utilization during an ED shift, some of which overlap with the barriers expressed by our faculty. Similar hurdles include time to perform bedside ultrasound, comfort with the machine, obtaining images, lack of training, and lack of understanding of how to document exams. Some authors also identified barriers not voiced in our study, including consultants requesting a comprehensive ultrasound performed by the radiology department, inability to locate machines, and high cost of equipment [15, 17, 18, 20]. Addressing these additional barriers should be considered in future workshops.

Future directions include launching a longitudinal certification program for EM physicians based on the “practice pathway” outlined by ACEP. This would include teaching and assessing core CUS knowledge, retention, and technical skill development to those EM faculty who desire further CUS training and/or certification. The same workshop format and course set-up could be used with additional CUS modalities such as lung, renal, ocular, and gallbladder ultrasound. Future workshops could also be adapted to other learner groups and include simulation stations to provide learners with a wider variety of anatomy and pathology.

Limitations

This study has several limitations. The population was a voluntary sampling of practicing EM physicians with varying levels of experience from an academic health system. This may introduce selection bias and limit the generalizability of the results. Despite this intervention improving physician comfort and reducing perceived barriers to CUS, it did not evaluate knowledge, retention, or ultrasound skill development. Ways to improve these limitations would be to augment this course with a structured skills workshop that assessed pre- and post-intervention knowledge and skills. In addition, providing follow-up opportunities to practice CUS in the clinical setting under instructor guidance could improve skill with the ultrasound machine, obtaining images, identifying normal anatomy versus pathology, and improving efficiency. Having additional CUS sessions or content boosters, such as ultrasound case conferences that focus on challenges and key concepts may help to build confidence and promote further interest in pursuing advanced skill development beyond this introductory workshop.

## Conclusions

Many EM physicians trained prior to the addition of ultrasound into residency curriculum and identify barriers to utilizing this technology in the clinical setting. Our CUS workshop fills an identified gap in ultrasound training by providing an effective method to expose seasoned physicians to core concepts of CUS through lectures and hands-on scanning sessions. After this 5-hour workshop, EM physicians felt more comfortable with ultrasound image acquisition and interpretation and identified fewer barriers to CUS utilization. This CUS workshop is an effective first step in the CUS training pathway.

## Figures and Tables

**Figure 1:**
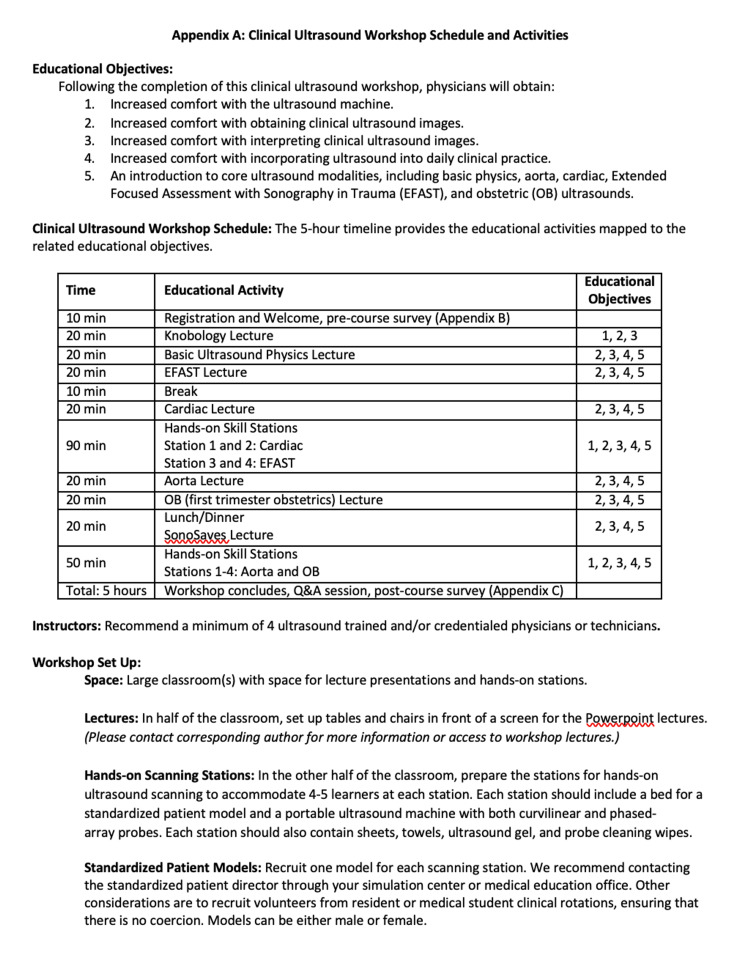
Appendix A: Clinical Ultrasound Workshop Schedule and Activities

**Figure 2:**
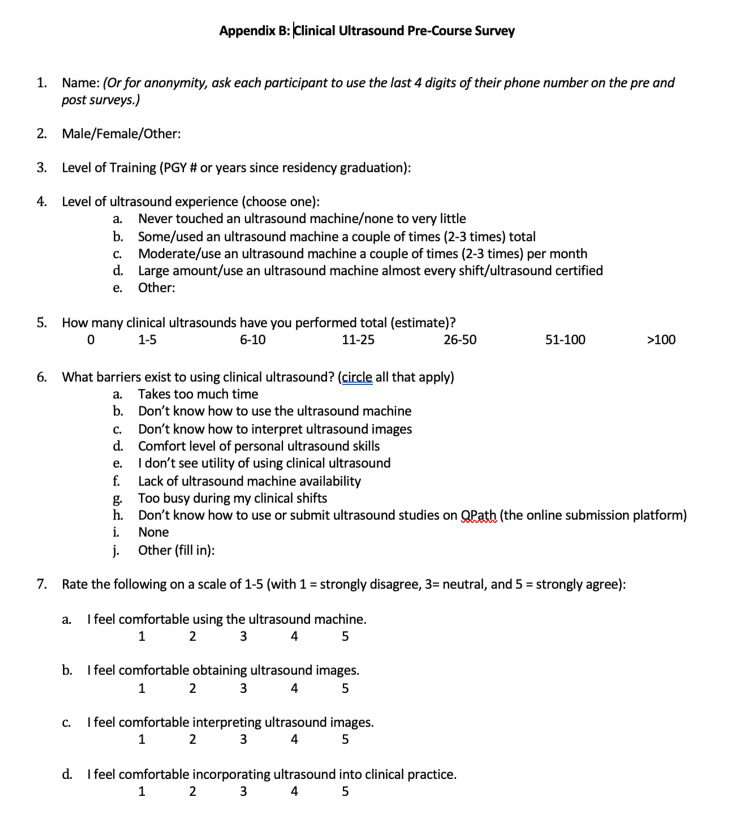
Appendix B: Clinical Ultrasound Pre-Course Survey

**Figure 3:**
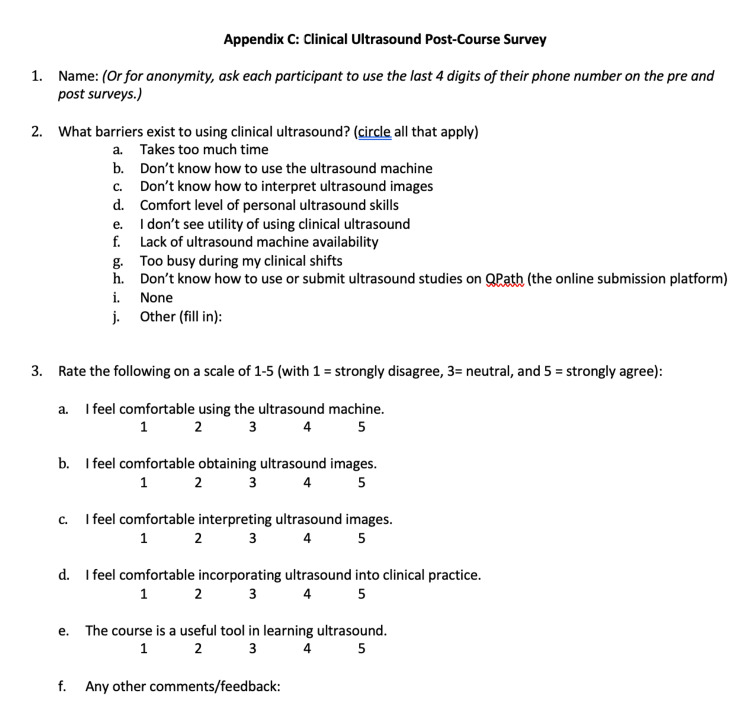
Appendix C: Clinical Ultrasound Post-Course Survey

## References

[1] Blaivas M, Harwood RA, Lambert MJ (1999). Decreasing length of stay with emergency ultrasound examination of the gallbladder. Acad Emerg Med.

[2] Blaivas M, Sierzenski P, Plecque D, Lambert M (2000). Do emergency physicians save time when locating a live intrauterine pregnancy with bedside ultrasonography?. Acad Emerg Med.

[3] Smith-Bindman R, Aubin C, Bailitz J (2014). Ultrasonography versus computed tomography for suspected nephrolithiasis. N Engl J Med.

[4] Jones AE, Tayal VS, Sullivan DM, Kline JA (2004). Randomized, controlled trial of immediate versus delayed goal-directed ultrasound to identify the cause of nontraumatic hypotension in emergency department patients. Crit Care Med.

[5] Melniker LA, Leibner E, McKenney MG, Lopez P, Briggs WM, Mancuso CA (2006). Randomized controlled clinical trial of point-of-care, limited ultrasonography for trauma in the emergency department: the first sonography outcomes assessment program trial. Ann Emerg Med.

[6] Howard ZD, Noble VE, Marill KA, Sajed D, Rodrigues M, Bertuzzi B, Liteplo AS (2014). Bedside ultrasound maximizes patient satisfaction. J Emerg Med.

[7] (2020). American College of Emergency Physician (ACEP) policy statement for ultrasound guidelines: emergency, point-of-care, and clinical ultrasound guidelines in medicine. https://www.acep.org/patient-care/policy-statements/ultrasound-guidelines-emergency-point-of--care-and-clinical-ultrasound-guidelines-in-medicine/.

[8] Hoffman T, Du Plessis M, Prekupec MP, Gielecki J, Zurada A, Tubbs RS, Loukas M (2017). Ultrasound-guided central venous catheterization: a review of the relevant anatomy, technique, complications, and anatomical variations. Clin Anat.

[9] Lewiss RE, Pearl M, Nomura JT (2013). CORD-AEUS: consensus document for the emergency ultrasound milestone project. Acad Emerg Med.

[10] Noble VE, Nelson BP, Sutingco AN, Marill KA, Cranmer H (2007). Assessment of knowledge retention and the value of proctored ultrasound exams after the introduction of an emergency ultrasound curriculum. BMC Med Educ.

[11] Schmid K (2020). Emergency ultrasound. EMRA Fellowship Guide, 2nd Edition.

[12] Moore CL, Molina AA, Lin H (2006). Ultrasonography in community emergency departments in the United States: access to ultrasonography performed by consultants and status of emergency physician-performed ultrasonography. Ann Emerg Med.

[13] Stein JC, River G, Kalika I, Hebig A, Price D, Jacoby VL, Filly R (2009). A survey of bedside ultrasound use by emergency physicians in California. J Ultrasound Med.

[14] Ahern M, Mallin MP, Weitzel S, Madsen T, Hunt P (2010). Variability in ultrasound education among emergency medicine residencies. West J Emerg Med.

[15] Lewiss RE, Saul T, Del Rios M (2013). Acquiring credentials in bedside ultrasound: a cross-sectional survey. BMJ Open.

[16] Shaffer M, Brown HA, McCoy C, Bashaka P (2017). Evaluation of a short-term training program in bedside emergency ultrasound in Southwestern Tanzania. J Ultrasound Med.

[17] Graglia S, Huang C, Shokoohi H, Liteplo AS (2019). Faculty opinions concerning ultrasound utilization in the emergency department. Am J Emerg Med.

[18] Wong J, Montague S, Wallace P (2020). Barriers to learning and using point-of-care ultrasound: a survey of practicing internists in six North American institutions. Ultrasound J.

[19] Maan ZN, Januszyk M, Rennert RC (2014). Noncontact, low-frequency ultrasound therapy enhances neovascularization and wound healing in diabetic mice. Plast Reconstr Surg.

[20] Schnittke N, Damewood S (2019). Identifying and overcoming barriers to resident use of point-of-care ultrasound. West J Emerg Med.

[21] Good R, Orsborn J, Stidham T (2018). Point-of-care ultrasound education for pediatric residents in the pediatric intensive care unit. MedEdPORTAL.

[22] Alkhalifah M, McLean M, Koshak A (2016). Acute cardiac tamponade: an adult simulation case for residents. MedEdPORTAL.

[23] Blackstock U, Carmody K (2016). Transforming learning anatomy: basics of ultrasound lecture and abdominal ultrasound anatomy hands-on session. MedEdPORTAL.

[24] Das D, Kapoor M, Brown C (2020). Comparison of hands-on versus online learning in teaching ultrasound skills for Achilles tendon rupture: a pilot study. Cureus.

[25] Russell FM, Herbert A, Zakeri B, Blaha M, Ferre RM, Sarmiento EJ, Wallach PM (2020). Training the trainer: faculty from across multiple specialties show improved confidence, knowledge and skill in point of care ultrasound after a short intervention. Cureus.

[26] Cassara M, Schertzer K, Falk MJ (2020). Applying educational theory and best practices to solve common challenges of simulation-based procedural training in emergency medicine. AEM Educ Train.

[27] Buscombe C (2013). Using Gagne's theory to teach procedural skills. Clin Teach.

[28] Frye AW, Hemmer PA (2012). Program evaluation models and related theories: AMEE guide no. 67. Med Teach.

[29] Shrestha R, Blank W, Shrestha AP, Pradhan A (2020). Evaluation of interdisciplinary emergency ultrasound workshop for primary care physicians in Nepal. Open Access Emerg Med.

[30] Smalley CM, Fertel BS, Broderick E (2020). Standardizing point-of-care ultrasound credentialing across a large health care system. Jt Comm J Qual Patient Saf.

